# Ion-Triggered In Situ Gelling Intranasal Spray of Dronedarone Hydrochloride Nanocarriers: In Vitro Optimization and In Vivo Pharmacokinetic Appraisal

**DOI:** 10.3390/pharmaceutics14112405

**Published:** 2022-11-08

**Authors:** Mahmoud H. Teaima, Doaa A. Helal, Jihad M. Alsofany, Mohamed A. El-Nabarawi, Mohamed Yasser

**Affiliations:** 1 Department of Pharmaceutics and Industrial Pharmacy, Faculty of Pharmacy, Cairo University, Cairo 11562, Egypt; 2 Department of Pharmaceutics, Faculty of Pharmacy, Fayoum University, Fayoum 63514, Egypt; 3 Department of Pharmaceutics and Industrial Pharmacy, Faculty of Pharmacy, University of Sadat City, 25th District, Sadat City 32897, Egypt; 4 Department of Pharmaceutics, Faculty of Pharmacy, Port Said University, Port Said 42526, Egypt; 5 Department of Pharmaceutics and Industrial Pharmacy, Faculty of Pharmacy, Horus University, New Damietta 34518, Egypt

**Keywords:** niosomes, in situ gel, gellan, nasal, dronedarone, bioavailability, pharmacokinetic

## Abstract

The current study aims to develop niosomal nanocarriers for intranasal delivery of dronedarone hydrochloride to ameliorate its limited bioavailability. Niosomes were prepared by ethanol injection method and optimized using 3² full factorial experimental design. Both Span^®^ type (X1) and Span^®^: cholesterol ratio (X2) were set as independent variables. Vesicle size (Y1), polydispersity index (Y2), zeta potential (Y3), and entrapment efficiency (Y4) were set as responses. The optimal formula was further incorporated into an ion-sensitive in situ gelling polymer for intranasal delivery. Optimal formula (N7), which is composed of Span^®^ 80: cholesterol (1:1), was of the least vesicle size (121.27 ± 13.31 nm), least polydispersity index (0.43 ± 0.073), highest zeta potential (−22.23 ± 2.84 mV) and highest entrapment efficiency (73.44 ± 2.8%). About 75.86% and 60.29% of dronedarone hydrochloride were released from N7 dispersion and in situ gel, respectively, within 12 h, compared to only 13.3% released from a drug-free suspension. In vivo pharmacokinetic study on male New Zealand rabbits resulted in significantly higher C_max_, AUC_0–72,_ and AUC_0–∞_ of intranasal niosomal in situ gel compared to oral suspension. Almost twofold amplification of relative bioavailability was obtained after intranasal administration of niosomal in situ gel (195.7%) compared to oral suspension.

## 1. Introduction

Dronedarone hydrochloride (DRN) is a non-iodine-containing benzofuran analog of the antiarrhythmic drug amiodarone, which is prescribed to cardiovascular patients with atrial fibrillation to lower the chances of hospitalization [1,2,3]. Dronedarone absorption is nearly 70–94% which increases significantly in the presence of food. However, the absolute bioavailability in the absence of food is reported as low as 4%, and this is mainly due to pre-systemic first-pass metabolism [4,5]. In the presence of fatty meals, the bioavailability of DRN increases to about 15%; hence, it is recommended to be taken with food [4,5]. Several gastrointestinal adverse events were reported upon the administration of oral DRN tablets, including stomach pain, indigestion, nausea, vomiting, heartburn, diarrhea, loss of appetite, and loss of taste [6]. Niosomes are amphiphilic nano-vesicular lipid carriers that are biodegradable, biocompatible, and nonimmunogenic. They are self-assembled upon mixing non-ionic surfactant with cholesterol in an aqueous medium [7]. Niosomes were first reported in the 1970s by researchers in the cosmetic industry [8]. The first niosome-based formulation was developed and patented by L’Oreal in 1975 [9]. Similar to their liposome precursors, the non-ionic nature of niosomes makes them excellent and effective drug delivery systems for both hydrophilic and lipophilic drugs [10]. Niosomes have been extensively investigated as a drug conveyor via different routes of administration such as oral [11,12], transdermal [13,14], intranasal [15], topical [16,17,18] and targeted drug delivery [19,20]. The intranasal route is an attractive direction for systemic drug administration. It is a surrogate pathway for drugs with deprived oral absorption and limited bioavailability resulting from gastrointestinal and/or hepatic metabolism [21,22]. The intranasal route is also accessible, safe, and non-invasive [15]. The abundance of blood vessels in the nasal mucosa contributes to better drug absorption, which is, at times, equivalent to intravenous injections [21]. However, drug absorption from the intranasal route is restricted by several factors, such as: the physicochemical properties of the drug [21], physiological and anatomical factors like nasal blood flow, enzymatic degradation, mucociliary clearance, and the physical condition of the nose [23]. Some pathological conditions of the nasal cavity, as well as natural mucociliary clearance, can reduce the capacity of nasal drug absorption, and the drug can be misplaced by dripping out of the nose or down the throat, thus reducing contact time and drug absorption by carrying the drug to the nasopharynx and then to the gastric intestinal tract. The inadequate contact time can be surmounted by incorporating the formulation into mucoadhesive polymers that can prolong drug residence at the absorption site and promote its absorption. These polymers can be exploited to formulate “in situ gels”, which have appropriate rheological behavior that allows them to flow upon administration (sol) and then to form (gel) under physiological conditions at the site of absorption. Gellan gum is a bacterial exo-polysaccharide formed by the bacteria *Sphingomonas elodea* [24]. Gellan gum is a promising polymer for intranasal drug delivery because of its capability to form a gel in situ upon contact with physiological cations [25]. DRN has been previously scrutinized for enhanced transdermal absorption by encapsulation into bilosomal vesicles [26] and oral absorption by implementation of different formulation technique such as proliposomes [27], self micro emulsifying drug delivery systems [5] and solid lipid nanoparticles [28]. As per authors’ knowledge, no former studies reported the bioavailability enhancement of DRN after intranasal administration so far.

The aim of work in the current study is to develop and optimize niosomal vesicles loaded with DRN to be delivered as an in situ gelling spray via intranasal mucosa to bypass first-pass metabolism, which occurs upon oral administration of the drug. Full factorial experimental design (3^2^) is to be implemented using Design Expert^®^ software. The independent variables are Span^®^ (Sp) type (X1) and Span^®^ to cholesterol (CH) ratio (X2), and the responses are vesicle size (VS); Y1, polydispersity index (PDI); Y2, zeta potential (ZP); Y3 and entrapment efficiency (EE%); Y4. The desirability function will be calculated to select the optimal formula of least VS, least PDI, highest ZP (absolute value), and highest EE%. The optimal formula of the highest desirability will be subjected to further characterization with respect to the release profile and thermal analysis (DSC) of the solid form after lyophilization. Transmission electron microscopy (TEM) will be implemented in the visualization of vesicle size and shape of the optimal formula in its liquid form. The physical stability of the optimal formula will be evaluated by reanalyzing VS, PDI, ZP, and EE% after storage for 90 days at 4 °C and at 25 °C. Optimal niosomes formula will be incorporated into ion-sensitive polysaccharide (gellan gum) that undergoes sol-to-gel phase transition upon intranasal administration. Gel formulation will be evaluated with respect to in situ gelation capability, pH, rheological properties, gel strength, mucoadhesion force, and release pattern. In vivo pharmacokinetic evaluation of the optimal niosomal intranasal in situ gel compared to oral drug suspension will be carried out on healthy male New Zealand rabbits after analysis of dronedarone concentration in rabbits’ plasma using a suitable valid bioanalytical method.

## 2. Materials and Methods

### 2.1. Materials

Dronedarone hydrochloride (DRN), purity: 100% *w*/*w* on anhydrous basis (Glenmark Generics Limited, Mumbai, India) was kindly gifted from Al-Andalus pharmaceutical company, Giza, Egypt. Cholesterol (CH) (Sigma Aldrich, St. Louis, MO, USA). Span^®^ 40 (SP40) (Oxford Lab Chem, Mumbai, India). Span^®^ 60 (SP60) (Oxford Lab Chem, Mumbai, India). Span^®^ 80 (SP80) (Oxford Lab Chem, Mumbai, India). Gellan gum (Alfa Aesar Company, Heysham, UK). Sodium chloride, potassium chloride, sodium phosphate dibasic, and Ethanol absolute (El Gomhoriyah chemical company, Cairo, Egypt). Cellulose dialysis membrane 12.000–14.000 M.wt cut off (SERVA Electrophoresis, Heidelberg, Germany). Nitrofurantoin Standard (Kahira Pharmaceutical and Chemical Industry Company, Cairo, Egypt). Formic acid (Merck, Kenilworth, NJ, USA). Acetonitrile (Supelco, Bellefonte, PA, USA), HPLC grade (Millipore Sigma, Burlington, MA, USA). HPLC grade water (Merck, Kenilworth, NJ, USA).

### 2.2. Preparation of DRN Loaded Niosomes

Niosomes were prepared by ethanol injection method [29]. Simply, drug (DRN) (100 mg/mL), Span (Sp), and cholesterol (CH) in different ratios (*w*:*w*), as described in Table 1, were dissolved in 10 mL ethanol. The resulting organic solution was gradually injected through fine injection needle (22 gauge size) connected to injection pump at fixed rate (20 drops/min) into 10 mL deionized water. The mixture was then magnetically stirred using hotplate magnetic stirrer (MS-H550-Pro, Medfuture Biotech CO, Jinan, China) at fixed rotation rate and maintained at 65 °C for 1 h. Magnetic stirring was continued until complete evaporation of ethanol to obtain drug-loaded niosomes. This dispersion was sonicated for 15 min using bath sonicator (Crest Ultrasonics Corp., Ewing Township, NJ, USA) to reduce vesicles size of any aggregates and stored at 2–8 °C until further use.

### 2.3. Characterization of the Prepared Niosomes

#### 2.3.1. Vesicle Size, Size Distribution, and Zeta Potential

Vesicle size (VS), size distribution represented by polydispersity index (PDI), and zeta potential (ZP) of the prepared DRN vesicles were measured using Zetasizer Nano ZS-90 instrument (Malvern Instruments, Southboro, MA, USA). The ZP analysis was determined by measurement of the electrophoretic mobility of the particles in colloidal dispersion [30]. An aliquot of the dispersion was diluted before the measurement. Measurements were performed in triplicate using 90° scattering angle at 25 °C, dispersant viscosity at 0.8872 cP, and dielectric constant at 78.5. The displayed results are the mean value ± SD.

#### 2.3.2. Entrapment Efficiency (EE%)

The EE% of DRN-loaded niosomes was determined indirectly by calculating the difference between the total amount of DRN incorporated into formulation and the amount that remained in the supernatant after separating the prepared vesicles by centrifugation at 15,000 rpm for 1 h at 4 °C using cooling centrifuge (Hermle Z326K, Labortechnik GmbH, Wehingen, Germany). The concentration of free DRN was measured spectrophotometrically by measuring the ultraviolet (UV) absorbance (Evolution 201 UV-visible spectrophotometer, Thermo Scientific, Waltham, MA, USA) at λ max 288 nm [31]. Drug EE% was calculated according to the following equation [32]:

(1) $$\mathrm{EE}\%=\frac{\mathrm{Total}\;\mathrm{amount}\;\mathrm{of}\;\mathrm{DRN}\;-\;\mathrm{Free}\;\mathrm{amount}\;\mathrm{of}\;\mathrm{DRN}}{\mathrm{Total}\;\mathrm{amount}\;\mathrm{of}\;\mathrm{DRN}\;}\times 100$$

### 2.4. Factorial Experimental Design of the Study

A 3^2^ full factorial experimental design was implemented to study the effect of different formulation variables on the prepared vesicles and to select the optimum conditions for niosomes formulation using Design Expert^®^ software (version 7, Stat-Ease, Inc., Minneapolis, MN, USA). Experimental trials were performed with all possible combinations for formulation of DRN-loaded niosomes. Design summary is represented in Table 1.

### 2.5. Selection and Validation of the Optimal DRN Loaded Niosomes Formula

The choice of optimal formula was based on the desirability function. The optimization recommended obtaining formula with the least VS, least PDI, highest EE%, and highest ZP absolute value.

#### 2.5.1. Transmission Electron Microscopy (TEM)

The optimized DRN-loaded niosomes formula was morphologically identified by TEM (JEOL RI 2100, Frankfurt, Germany). A drop of freshly prepared DRN niosomal dispersion was diluted and stained with phosphotungstic acid 0.01% (*w*/*v*) and placed on a copper grid. The copper grid was fixed into sample holder and placed in the vacuum chamber of the electron microscope. Images were captured using small and large magnifications.

#### 2.5.2. Lyophilization of the Optimized DRN Loaded Niosomes

The optimized formula was lyophilized after preparation. First, DRN niosomes dispersion was stored in glass flasks and pre-frozen at −80 °C for 24 h (ultra-low temperature freezer, So-low, Ultra-Low Freezer, Environmental Equipment, Cincinnati, OH, USA), and the samples were freeze-dried using freeze dryer (Bondiro Freeze Dryer, IIshin Lab Co Ltd., Yangju, Korea) at −90 °C and 380 mT of pressure for 48 h to yield dry powder.

#### 2.5.3. Differential Scanning Calorimetry (DSC)

Thermal analysis of 5 mg from each sample of pure DRN and lyophilized optimal formula was carried out using DSC (DSC-60, Shimadzu Corp., Kyoto, Japan) in a temperature range of 10–400 °C at a scanning rate of 10 °C/min under inert nitrogen flow (25 mL/min) standardized with purified indium. Samples were weighed and encapsulated in flat-bottomed aluminum pans with crimps-on lids for analysis. The incompatibilities and/or crystallinity state were detected by shifting or disappearance of the corresponding peaks [33].

### 2.6. Stability Study of the Optimal DRN Loaded Niosomes Formula

The optimal DRN-loaded niosomes formula was stored at 4 °C and 25 °C for 90 days. Samples from each formulation were taken at 0 and 90 days. Stability was anticipated by assessment of VS, PDI, ZP, and EE% (measurements were carried out as mentioned earlier) at zero time relative to results obtained after 90 days of storage [34]. Statistical significance was analyzed by ANOVA test using SPSS^®^ software version 17.0 (SPSS^®^, Chicago, IL, USA). Difference at *p* ≤ 0.05 was considered significant.

### 2.7. Preparation of Niosomal Ion Sensitive In Situ Gelling Intranasal Spray

An accurately weighed quantity of gellan gum (0.5%, 0.75%, 1.0% *w*/*v*) was dissolved in 10 mL of pre-heated deionized water (90 °C) with continuous stirring till the polymer dissolved completely, and homogenous dispersion was obtained. Subsequently, optimal niosomal formula was added to the gellan aqueous dispersion in ratio (1:1) and mixed thoroughly till homogeneity. The prepared DRN niosomes–gellan mixture was denoted as (sol). Finally, the prepared formulations were filled in metered pump nasal spray containers.

### 2.8. Evaluation of DRN Niosomal In Situ Gelling Intranasal Spray

#### 2.8.1. Assessment of In Situ Gelation Capability

The capability of in situ gelations of the prepared polymeric niosomal spray (sol) formulations was visually evaluated in vitro. Specific volume from each formula (sol) was added into a transparent glass vial containing an equal volume of simulated nasal fluid solution (SNF) pH 5.5 at 32 °C (temperature of nasal mucosa 30–34 °C). The vial was then inverted after 1 min to judge the gelation capability. The resulting gel was observed and denoted (gel). Furthermore, in situ gelations of sol formulations was confirmed by spraying each formulation on a filter paper soaked with SNF. Simulated nasal fluid was prepared by dissolving 7.45 g/L NaCl, 1.29 g/L KCl and 0.32 g/L CaCl_2_.2H_2_O in distilled water, and the pH was adjusted to 5.5.

#### 2.8.2. Measurement of pH

The pH of the DRN niosomal in situ gel was determined using a pH meter (MEDFUTURE 920 precision PH/ORP meter, Medfuture Biotech CO, Jinan, China) at 25 °C. All measurements were performed in triplicate, and the final value was represented as mean value ± SD.

#### 2.8.3. Assessment of Rheological Properties

The viscosity values of the prepared N7-gellan polymeric mixture (both sol and gel phases) were measured with a rotational type rheometer (Brookfield Digital Rheometer type DV III, Brookfield Engineering, Middleboro, MA, USA). The shear stress formed under different shear rate conditions (Speed of rotation was changed between 5 and 25 rpm and in reversed order) using CPE-40 spindle. The temperature was set to 25 ± 2 °C, and the viscosity values of gel were measured. The rheological parameters (viscosity, shear rate, and shear stress) were obtained [35]. Finally, the flow properties of gel were determined by plotting the shear rate against the shear stress. The obtained data were fitted to the power-law model represented below to predict the rheological behavior of the formed gel:

τ = *k*γ^𝓃^ (2)

where τ is the shear stress (dyne/cm^2^), *k* is the consistency index (dyne/cm^2^ sn^2^) and γ is the shear rate (s^−1^) and 𝓃 is the index of flow.

#### 2.8.4. Evaluation of Gel Strength

Gel strength was determined by measuring the force required to lower a distance of 4 mm from the surface of the gel using a standard cylinder probe with diameter of 12.7 mm using texture analyzer (TA.XT Plus with Exponent software^®^ version 6, Stable micro systems Ltd., Godalming, UK).

#### 2.8.5. Evaluation of Mucoadhesion Force

Texture analyzer (TA.XT plus Texture Analyser, Stable micro systems Ltd., Godalming, UK)was used for the mucoadhesion measurement using accessory intended for measurement of the mucoadhesion force of gel i.e., mucoadhesion prope (TA.XT plus Texture Analyser, Stable micro systems Ltd., Godalming, UK). The force required to detach the gel from the surface of mucin compressed tablet using 5 kg load cell was determined. Mucin tablet was hydrated with moistened tissue and fixed to the moving upper probe of texture analyzer using double-face adhesive tape. Gel was placed in a plate and maintained at 37 ± 2 °C on the lower stage of texture analyzer. The tablet was lowered on the surface of the gel and left in contact for 30 s. Analysis was performed at the specifications of pre-test speed of 0.5 mm/s, test speed of 0.5 mm/s, post-test speed of 0.5 mm/s, applied force of 0.2 N, contact time of 30 s, return distance of 10 mm, and acquisition rate of 500 pps. Trigger type was set to auto-0.05 N while tare mode was set to auto with the option of return to start. The probe was then moved vertically upward after keeping in contact for 30 s. Mucoadhesion force was measured in triplicate and results represented as mean ± SD.

#### 2.8.6. In Vitro Release Study of Niosomal Dispersion and Niosomal In Situ Gel

DRN release from niosomal dispersion and niosomal in situ gel vs. drug-free suspension was scrutinized. Amount of drug-free suspension, DRN niosomal dispersion, and DRN niosomal sol equivalent to 20 mg DRN were used in release study. For niosomal sol, an equivalent volume of SNF was added and mixed with niosomal dispersion to form in situ gel in plastic cylindrical tubes with fixed permeation areas that have one end tightly enclosed with a dialysis membrane (membrane was immersed overnight in release medium before experiment). The other end connected to the shaft of the USP dissolution apparatus I (COPLY Dissolution Tester, Copley Scientific Limited, Nottingham, UK) substituting baskets’ position for 12 h at 37 ± 2 °C. Tubes containing gel were immersed in 100 mL SNF pH 5.5 containing alcohol (3:2) to maintain sink condition. Samples of niosomal dispersion and drug suspension were separately added to similar tubes and connected to rotating dissolution shaft substituting baskets’ position. Rotation speed was set to 100 rpm. Sampling was performed every 1 h time interval and up to 12 h. Samples were measured spectrophotometrically at λ max 288 nm. Release profiles of tested samples were represented by plotting the% cumulative amounts of DRN released against time (h). Similarity factor (ƒ_2_) was calculated to compare the release profile of the optimal niosomal dispersion and niosomal in situ gel to that of the drug-free suspension according to the following equation [36]:

ƒ_2_ = 50 × log 10 [(1 + W/n)^−1/2^ × 100] (3)

where: W is the sum of squares of differences in average cumulative percent DRN released between the formulations in comparison to overall sampling time points, and n is the number of sampling time points. Experiments were performed in triplicate, and results are represented as mean ± SD.

#### 2.8.7. Release Mechanism of Optimal DRN Niosomal In Situ Gel

Release data were fitted in different mathematical kinetic models [37,38] such as Zero-order (% of DRN release vs. time), First-order (log of% DRN remaining vs. time), Higuchi equation (% of DRN release vs. square root of time), Korsmeyer–Peppas equation (log of % DRN release vs. log time) and Hixson–Crowell equation (cube root of % DRN released vs. time) to predict the mechanism of DRN release from niosomal in situ gel. For each model, regression coefficient (R^2^) value was determined. The regression coefficient R^2^ value nearer to 1 indicated that the model fits the release mechanism. In the Korsmeyer–Peppas model, the “n” value obtained from the slope of the plot was applied to characterize the DRN release mechanism as described below:

- n < 0.5 (0.45)—quasi-Fickian diffusion.
- n = 0.5 (0.45)—diffusion mechanism.
- 0.5 (0.45) < n < 1—non-Fickian diffusion.
- n = 1 (0.89)—case II transport (zero-order release).
- n > 1 (0.89)—super case II transport.

### 2.9. In Vivo Pharmacokinetic Assessment

#### 2.9.1. Animal Study Design and Samples Collection

Two groups of healthy New Zealand male white rabbits (2–3 kg) were employed in the in vivo pharmacokinetic assessment, with 4 rabbits in each group. The in vivo pharmacokinetic study was conducted according to the guidelines of the Declaration of Helsinki and approved by the Research Ethics Committee of Faculty of Pharmacy Cairo University, Egypt (serial number of protocol: PI 2785 valid from 26 October 2020). The utilization and handling of animals complied with the EU directive 2010/63/EU. The animals were housed in laboratory in accordance with the Guide for the Care and Use of Laboratory Animals [39] at well-defined and controlled conditions of 25 ± 2 °C temperature, 57 ± 10% humidity, and 12 h light-dark cycle and had free access to water and food. The animals were randomly divided into two groups; each group included four animals. A single-dose, parallel design was followed in this study. The utilized DRN dose was adjusted to be (20 mg/kg) based on rabbits’ body surface according to the following equation [40]:

(4) $$\mathrm{Human}\;\mathrm{equivalent}\;\mathrm{dose}=\mathrm{animal}\;\mathrm{dose}\left(\mathrm{mg}/\mathrm{kg}\right)\times \frac{\mathrm{animal}\;\mathrm{Km}}{\mathrm{human}\;\mathrm{Km}}$$

where: Km = Body weight (kg)/Body surface area (m^2^). The first group received DRN niosomal gellan sol via intranasal route (250 µL of sol in each nostril) using micro injector fitted with soft polyethylene tube of 0.10 mm internal diameter (treatment N). The second group received 5 mL of aqueous drug suspension (prepared by sprinkling the required drug dose in 5 mL deionized water with vigorous shaking) by oral gavage (treatment O). Blood samples (2 mL) were withdrawn by retro-orbital plexus puncture in heparinized eppendorf tubes after 0, 0.5, 1, 2, 3, 4, 5, 6, 8, 12, 18, 24, 36, 48, 60, and 72 h post-administration. Centrifugation of blood was performed immediately at 3000 rpm for 15 min (Hanil Cooling Centrifuge, Hanil Scientific Inc, Gimpo, Korea). The plasma was separated and stored at −20 °C for further analysis.

#### 2.9.2. Bioanalytical Method Description and Chromatographic Conditions

Plasma samples were analyzed using a sensitive, reproducible, and accurate Liquid Chromatography/Mass Spectrometry (LC-MS/MS) method (Agilent HPLC-MS/MS, Agilent Technologies Inc, Santa Clara, CA, USA), developed and validated before the study in accordance with the international guidelines. The isocratic mobile phase used was a mixture of 0.1% formic acid in Acetonitrile: 0.1% formic acid in water (45:55% *v*:*v*) which was delivered using Agilent eclipse plus C18 (4.6 × 50 mm, 3.5 µm) column at a flow rate of 0.7 mL/min and 30 °C temperature and with an injection volume of 10 µL and sampler temperature of 15 °C into the mass spectrometer’s electrospray ionization chamber. The mass spectrometric conditions were set as described in Table 2. The analytical data were processed using MassHunter^®^ version 10 software.

#### 2.9.3. Plasma Samples Preparation

All frozen rabbits’ plasma samples were thawed at ambient temperature. A total of 30 µL from internal standard (IS) solution (50 µg/mL nitrofurantoin) was spiked into 300 µL of plasma samples, vortex for 1 min (Multi-tube Vortex Mixer, Paramix, Julabo Laboratechnik GmbH, Seelbak, Germany). Protein precipitation was carried out by addition of 1 mL acetonitrile. The samples were vortex-mixed for 3 min and centrifuged at 4000 rpm for 5 min at 4 °C. Then the clear supernatant was analyzed by LC-MS/MS system. The concentration of dronedarone was plotted against time and analyzed by Winnonlin Phoenix^®^ version 4.3.4 software using non-compartmental pharmacokinetic model. For single-dose studies, pharmacokinetic analysis includes calculation of the following pharmacokinetic parameters [41]:

C_max_, T_max,_ AUC_0–72,_ AUC_0–∞_, t_½_ and K_el_. Elimination half-life (t_1/2_) (h) was calculated as 0.693/K. The maximum drug concentration (C_max_, ng/mL) and the time of its occurrence (T_max_, h) were acquired from the individual plasma concentration time curves. The area under the curve (AUC_0–72_) (ng h/mL) was calculated by the linear trapezoidal rule. The area under the curve from zero to infinity (AUC_0–∞_) (ng h/mL) was calculated as AUC_0–∞_ = AUC_0–72_ + C_t_/K, where C_t_ is the last measured concentration at the time t. C_max_, t_1/2_, AUC_0–72_, and AUC_0–∞_ were compared between the two treatments utilizing ANOVA test for the untransformed data. Furthermore, the non-parametric signed-rank test (Mann–Whitney’s test) was employed for the comparison of medians of T_max_ for both treatments using SPSS^®^ software [41]. Difference at *p* ≤ 0.05 was considered significant. The relative bioavailability of intranasal DRN niosomal in situ gel compared to the free DRN oral suspension was calculated using the following equation [41]:

(5) $$\%\;\mathrm{Relative}\;\mathrm{bioavailability}\;\mathrm{of}\;\mathrm{intranasal}\;\mathrm{DRN}\;\mathrm{niosomal}\;\mathrm{in}\;\mathrm{situ}\;\mathrm{gel}\;=\frac{{\mathrm{AUC}}_{0–\infty }\;\left(\mathrm{intranasal}\;\mathrm{DRN}\;\mathrm{niosomal}\;\mathrm{in}\;\mathrm{situ}\;\mathrm{gel}\right)}{{\mathrm{AUC}}_{0–\infty }\left(\mathrm{DRN}\;\mathrm{oral}\;\mathrm{suspension}\right)}\times 100$$

## 3. Results and Discussion

### 3.1. Analysis of 3^2^ Full Factorial Design

**Figure 1:**
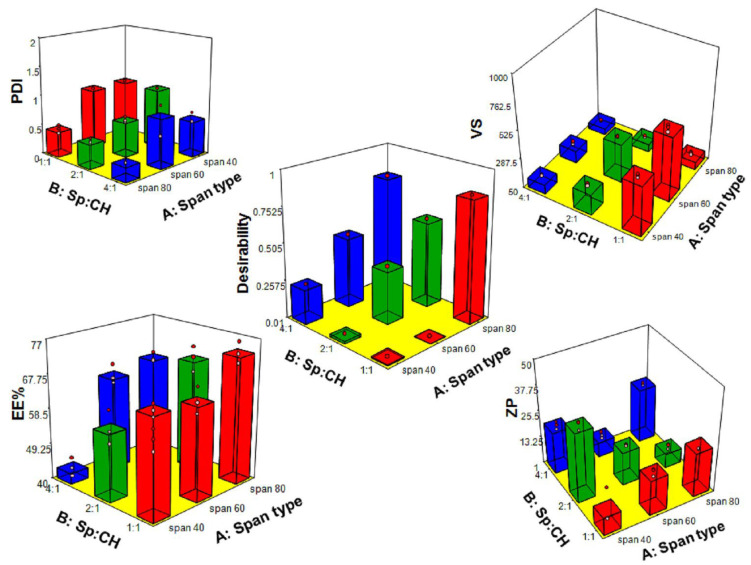
Three-dimensional Response plot for the effect of Span^®^ type (X1) and Span^®^: cholesterol ratio (Sp:CH) (X2) on vesicle size (VS), polydispersity index (PDI), zeta potential (ZP), entrapment efficiency (EE%) and desirability of DRN loaded niosomes.

Factorial designs allow concurrent investigation of the impact of different formulation variables on the features of the drug formulation. Table 3 summarizes the combination of variables in each experimental run and the corresponding responses. Fit statistics analysis was performed for each response individually to obtain a polynomial model describing the relation between this response and the studied variables. The software suggests the best fitting model for every response based on maximizing the Adjusted R^2^ and the lowest predicted residual error sum of squares. According to the model fit statistics presented in Table 1, the suggested model was 2-factor interactions (2FI) for the four responses. The predicted R^2^ reasonably coincides with the adjusted R^2^ (the difference is less than 0.2) for all responses demonstrating the model’s suitability. Additionally, an adequate precision of more than four indicates an appropriate signal-to-noise ratio. Therefore, the 2FI model was verified to be suitable for the investigation of the experimental design space. The desirability function is a useful tool to identify the optimal levels of the tested variables. Design 3^2^ statistics are summarized in Table 1. The results of the 3^2^ factorial design analysis are illustrated in Figure 1. The optimal formula of the highest desirability was chosen based on preset criteria of small VS as smaller size vesicles can easily traverse nasal mucosa and be well absorbed, small PDI as low values of PDI ensure physical stability and homogeneity of the formed vesicles, the high absolute value of ZP, which also indicates good stability of the formed vesicles, and high EE%, which assure that almost all the dose is encapsulated inside the vesicles which verify the successfulness of formulation to improve drug properties.

#### 3.1.1. Effect of Variables on VS

Vesicle size is a fundamental aspect of nanocarriers’ formulation and optimization. Smaller vesicles have a higher potential to pass through nasal mucosa into the capillary network. Vesicle size measured for the prepared DRN-loaded niosomes ranged from 105.87 ± 6.71 nm for N9 to 607.643 ± 29.5 nm for N4. Results are represented in Figure 1 and Table 3. ANOVA testing of VS model revealed that both of the examined factors (X1 and X2) were significant (*p* < 0.05). Considering Span^®^ type (X1), niosomes prepared by Sp80 had the smallest vesicle size. In this study, VS decreased in the following order: Sp60 > Sp40 > Sp80. The results revealed that the size of niosomes tended to increase with a progressive increase in the HLB value of Span^®^ used in the formulation. The smallest average size (105.87 ± 6.71 nm) was observed in the case of Sp80 (HLB 4.3) based vesicles (N9). Vesicles of larger size (607.643 ± 29.5 nm) were obtained in the case of Sp60 (HLB 4.7) based niosomes (N4). This might be due to surface-free energy as it decreases with increasing hydrophobicity [42]. Khazaeli and Pardakhty [43] reported that niosomes composed of sorbitan monoesters (Sp20, Sp40, and Sp60) were relatively larger in size as compared to Sp80-based niosomes. Similar results were reported for Sp85 (HLB 1.8) niosomes, which showed a smaller size compared to Sp20-, Sp40-, Sp60-, and Sp80-based niosomes [42]. Ruckmani et al. [44] showed that niosomes consisting of Sp60, T80, and T20 were larger in size, while in the case of Sp80 niosomes, the average size measured was relatively smaller, which may be due to lower HLB value. When the HLB value increases, the water uptake by the formed vesicles also increases, which results in larger size vesicles [45]. Gupta et al. also reported similar results in their work on fluconazole niosomes [46]. Regarding Sp: CH ratio (X2), increasing CH concentration resulted in increased VS. It was reported that a high level of CH hinders the close packing of vesicles’ elements, resulting in an improved distribution of aqueous phase within the niosomal vesicle, accordingly, increases VS [47,48]. Furthermore, the increase in CH concentration was correlated to the increased drug load within the niosomes, which might have as well contributed to the increase in the size of the vesicles. There is a correlation between VS and the amount of drug encapsulated inside vesicles as the space between the niosome bilayers increases upon drug inclusion in the hydrophobic domain within the vesicles [49].

#### 3.1.2. Effect of Variables on PDI

A lower PDI value is a good sign of physical stability, as higher values provoke Ostwald ripening [41,50]. The higher the PDI values, the lower uniformity of the vesicles in dispersion, whereas values closer to zero correspond to more homogenous niosomal dispersion [51]. In our study, PDI values of all formulations oscillated between 0.25 ± 0.01 for N9 and 1 ± 0.00 for N1, N2, and N4 (Table 3). PDI is preferred to be less than 0.3, but values within the 0.3 and 0.7 range are still acceptable because different size distribution algorithms work with values in the range of 0.05–0.7 [52]. ANOVA testing of the PDI model verified that both variables significantly affected PDI values (*p* < 0.05). Regarding Span^®^ type (X1), Sp80 resulted in the lowest PDI compared to other types of Span^®^ used. Lower PDI is attributed to the lower VS of niosomes prepared using Sp80. A similar result was obtained by Almahallawi et al. [53] in their work on tenoxicam bilosomes. Increasing the Sp:CH ratio (X2) resulted in decreased PDI values. High CH content might be the reason behind the increased drug loaded inside the vesicles, and thereby, heterogeneous vesicles were obtained with larger PDI. A similar finding was also reported by Aithal et al. [54], who confirmed an increase in PDI upon increasing the CH content of liposomes.

#### 3.1.3. Effect of Variables on ZP

The measured ZP values of niosomes ranged from −7.85 ± 1.2 mV to −34.3 ± 2.03 (Table 3), demonstrating that some niosomes formulations had enough charges that would hinder their aggregation and improve nano-vesicles stability. The lower negative values of zeta potential of the other formulations in this study were due to the non-ionic nature of the used surfactants (Spans^®^). Non-ionic surfactants stabilize niosomes mainly by steric hindrance. However, electrostatic stabilization may also occur. The negative charge of prepared niosomes might originate from the possible partial hydrolysis of the hydrophilic head of Spans^®^ surfactants, which are normally directed towards an aqueous medium [55] and/or negative charge carried on the hydroxyl group of cholesterol molecule in an aqueous medium. Both steric and electrostatic mechanisms of stabilization may operate simultaneously, particularly in aqueous systems. Steric stabilization is more advantageous in aqueous systems owing to intolerance to the added electrolytes; thus, stable dispersions could be prepared at much higher solid concentrations. Span^®^ type (X1) was the only significant model term (*p* < 0.05). Vesicles prepared by Sp80 showed significantly higher ZP values than those prepared using Sp60 or Sp40. This may be attributed to the unsaturation present in the hydrocarbon skeleton of Sp 80, which augmented the values of ZP contrary to vesicles prepared with the surfactants of saturated alkyl chains (Sp 40 and Sp60). It was previously reported that the higher the degree of unsaturation, the higher the zeta potential value [56].

#### 3.1.4. Effect of Variables on EE%

The obtained EE% of the nine formulations oscillated between 42.96 ± 2.58% for N3 and 73.44 ± 2.8% for N7 (Table 3). The reasonably high EE% can be explained by the high affinity of the highly lipophilic DRN (logP = 6.46) [4] to the lipids of the niosome membranes. Additionally, the low hydrophilicity of the utilized non-ionic surfactant (HLB values of Sp80 = 4.3, Sp60 = 4.7, Sp40 = 6.7) participated in the efficient entrapment of lipophilic DRN into niosomes [46]. ANOVA test revealed that both of the examined variables significantly affected EE%. Considering Span^®^ type (X1), Sp80 resulted in higher DRN entrapment than either Sp60 or Sp40. This could be attributed to the chemical structure of the investigated surfactants. Surfactants with longer alkyl chains (Sp80 (C18), Sp60 (C18) and Sp40 (C16)) could have resulted in higher entrapment efficiency [46,57]. In addition, being more hydrophobic, Sp80 (HLB 4.3) increased the affinity of the utilized surfactant to the hydrophobic DRN. For Sp: CH ratio (X2), higher CH concentration resulted in higher EE%. This may be due to the high affinity of lipophilic DRN to the lipids (CH) present in niosomes.

### 3.2. Selection and Validation of the Optimal DRN Loaded Niosomes

The optimal formula from the 3^2^ factorial design of the highest desirability value (0.837) was N7, which accomplished the preset criteria of small VS, small PDI, high ZP, and high EE% (Table 3). N7 was chosen as the optimal formula for further studies.

#### 3.2.1. Transmission Electron Microscopy (TEM)

**Figure 2:**
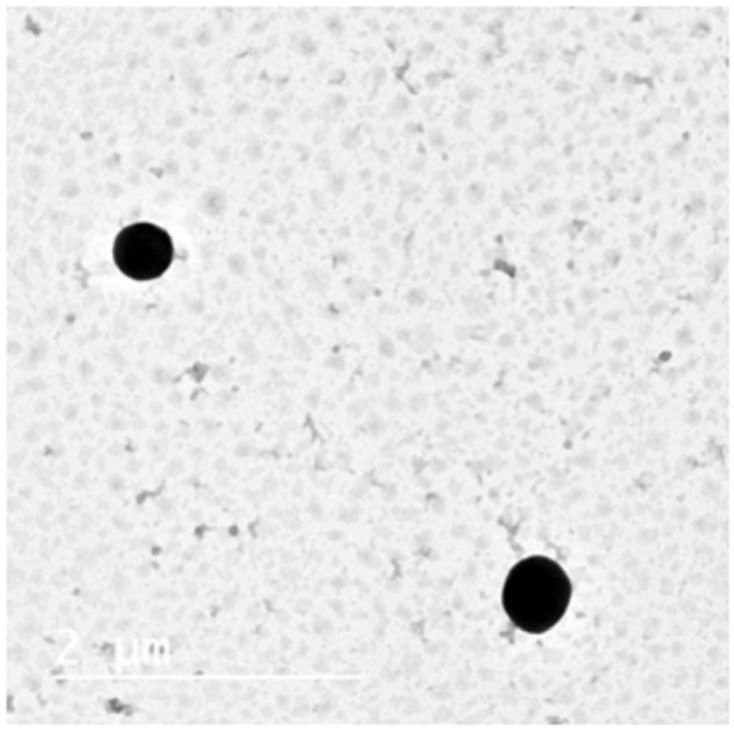
TEM micrograph of the optimized DRN loaded niosomes dispersion (N7).

The TEM micrograph represented in Figure 2 confirmed the spherical shape of niosomes vesicles without any irregularities, smooth surface, and narrow size distribution. Vesicle size observed by TEM was in good agreement with that measured by dynamic light scattering using Zetasizer.

#### 3.2.2. Differential Scanning Calorimetry (DSC)

**Figure 3:**
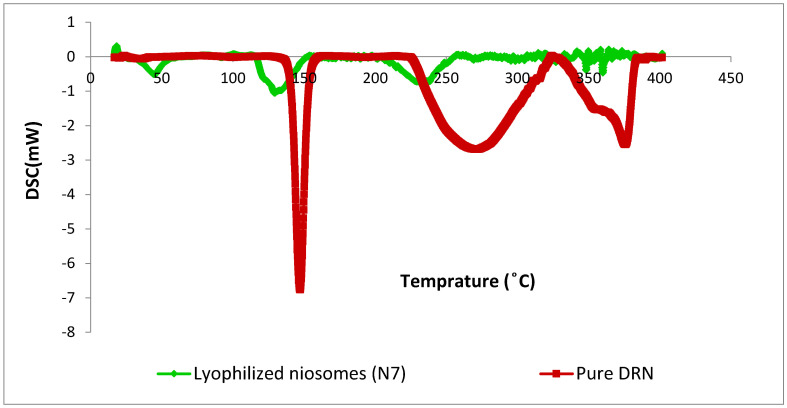
Thermograms of pure DRN and lyophilized optimal DRN loaded niosomes (N7).

The thermograms of pure DRN and lyophilized N7 are illustrated in Figure 3. Pure DRN showed a characteristic and distinctive endothermic peak at 146.30 °C, corresponding to its melting point, which suggests its purity and crystallinity. The enthalpy was found to be −309.88 mJ/g. The DSC curve of DRN in lyophilized N7 was observed at 129.48 °C corresponding to its melting point, and the enthalpy was found to be −135.81 mJ/g. The characteristic endothermic peak, corresponding to pure DRN melting, was shifted towards a lower temperature with lower peak intensity. The lower intensity of the DRN peak in lyophilized N7 may be due to the dilution of DRN in the excipients used. Furthermore, the reduced intensity and broadening of DRN peaks, which was reallocated towards lower temperature, might be due to the decrease in DRN crystallinity upon entrapment into niosomes in addition to possible solid-state interaction induced by heating.

### 3.3. Stability Study of the Optimal DRN Loaded Niosomes Formula

After 90 days at 4 °C and 25 °C storage conditions, no visible aggregation or change was detected in stored niosomes (N7). Retested VS, PDI, ZP, and EE% showed no significant differences from freshly prepared samples of N7. The stability results are summarized in Table 4. The negative charge of ZP enhanced the ZP values and prevented vesicle aggregation. The presence of non-ionic surfactant (Sp 80) could impart certain steric hindrance at the niosomes membrane surface, which prevented their aggregation and maintained their stability.

### 3.4. Evaluation of DRN Niosomal In Situ Gel

#### 3.4.1. Assessment of In Situ Gelation Capability

Gelation capability is one of the prerequisites for the successful formulation of in situ gels. When applied to the nasal cavity, DRN niosomes–gellan mixture (sol) undergoes a sol-gel phase transition due to ionic cross-linking interactions provoked by ions of physiological nasal fluid. The gelation concentration of gellan was determined according to the minimal concentration that could produce a persistent gel of suitable viscosity. Formula prepared with 0.5% gellan gum showed no spontaneous gelling upon contact with SNF, whereas formula containing 0.75% gellan resulted in flowing viscous liquid upon mixing with SNF. Only the formula prepared with 1% gellan showed spontaneous gel formation upon contact with SNF, which maintained its consistency upon storage for several days at room temperature. Gellan exhibits a coil-helix conformational change in the presence of low molecular weight salts accompanied by gelation due to the formation of double helices [58].

#### 3.4.2. Measurement of pH

The measured pH value for the N7 in situ gel was 5.13 ± 0.3, which was within the required pH range for nasal formulations (4.5–6.5). This suggests that the formula is convenient for intranasal administration.

#### 3.4.3. Viscosity Measurements and Rheological Properties

**Figure 4:**
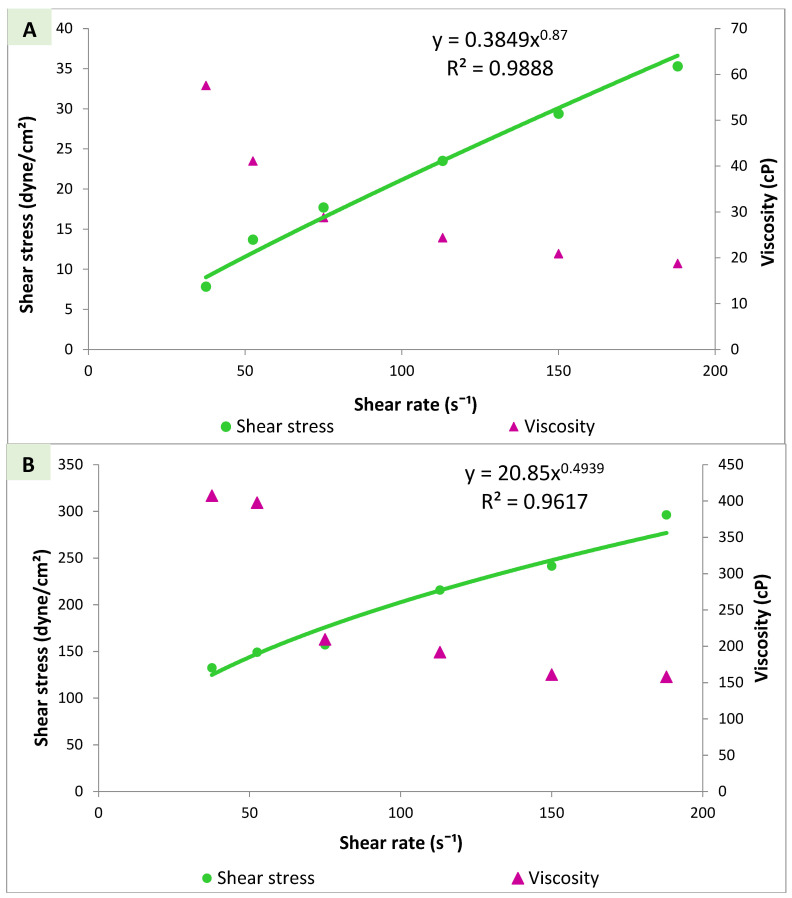
Rheogram of N7-gellan mixture ((**A**) sol phase, (**B**) gel phase).

The rheological properties of in situ gels for intranasal absorption are extremely imperative because the anatomy of the nasal cavity restricts conventional nasal formulation adherence to this region. In situ gelling intranasal formulations readily reach nasal mucosa and transform into a stagnant gel in the presence of electrolytes of nasal fluid, which provides an extended retention time and enhanced bioavailability [59]. The rheogram of sol and gel phases of the N7-gellan polymeric mixture is represented in Figure 4. Both sol and gel demonstrated non-Newtonian pseudoplastic shear thinning flow that was confirmed by flow index value (𝓃) lower than 1 and by viscosity decrease upon increasing shear rate [35]. The lower the value of (𝓃), the more shear thinning of the formulation [60]. Psuedoplastic flow allows easy dispensing of nasal formulation from a spray pump container after gentle shaking (sol). After application into the nasal cavity, a pseudoplastic system exhibits an increase in viscosity once in contact with the ions in the nasal fluid (gel), thus prolonging the nasal residence time [59]. This type of flow is preferred to maintain formula stability and prevent vesicle aggregation upon storage, allow easy dispensing of the spray upon shaking the container (sol), and restore gel viscosity and retention after nasal application to permit gel adhesion and retention on the nasal mucosa. In summary, in situ gelling inside the nasal cavity resulted from two synergistic aspects, i.e., the in situ gelling property of gellan, which was triggered by nasal fluid ions and the psuedoplastic flow of the applied spray which exist as a viscous liquid inside the container then turns into thin fluid upon pumping and shaking during application and finally transformed into gel inside the nasal cavity.

#### 3.4.4. Evaluation of Gel Strength

The gel strength value of N7 in situ gel was 123.80 ± 14.63 g. This relatively high value suggests the rigidness of the in situ gel formed upon contact with nasal fluid, which is a demand to enhance the retention of the formula in the nasal cavity and to decrease the drainage from the nose after dose application. An ideal in situ gel should easily maintain its strength rather than eroding or dissolving and hold the drug at the absorption site for an extended duration [61].

#### 3.4.5. Evaluation of Mucoadhesion Force

The measured mucoadhesion force of N7 in situ gel was 0.1 ± 0.026 N. The obtained quite high value of mucoadhesion is predictable as polymers containing the carboxylic group exhibit the best mucoadhesive properties [36]. This relatively high value of mucoadhesiveness is beneficial as it ensures better adhesion at the mucosal surface and, thereby, lengthens retention time and improves therapeutic efficacy [59]. Mucoadhesion is a complex process involving several concomitant interfacial interactions with the biological surface, particularly with mucin molecules that construct the mucus layer nearby the epithelium. Such interactions can occur by diverse mechanisms such as adsorption, diffusion, dehydration, electrostatic interaction, mechanical entrapment, and humidification [58,62]. The mucoadhesive character exhibited by the gellan gum resulted from the interaction of the specific functional groups in the polymer-like hydroxyl and carboxyl groups with the glycoprotein chain in the mucous layers of epithelial cells of the nasal cavity [58,62,63]. The interaction of the polymer with the glycoprotein chains in the mucin network occurs when the polymer swells by absorbing water from the mucous layer, and adhesion binding takes place to form gel [58,62,63]. The key factors that control the mucoadhesion process are the physiological ions in the nasal cavity and the swelling capacity of the polymer, which eventually lead to prolonged mucoadhesion and decreased mucociliary clearance [64].

### 3.5. In Vitro Release Study of Niosomes Dispersion and Niosomal In Situ Gel

**Figure 5:**
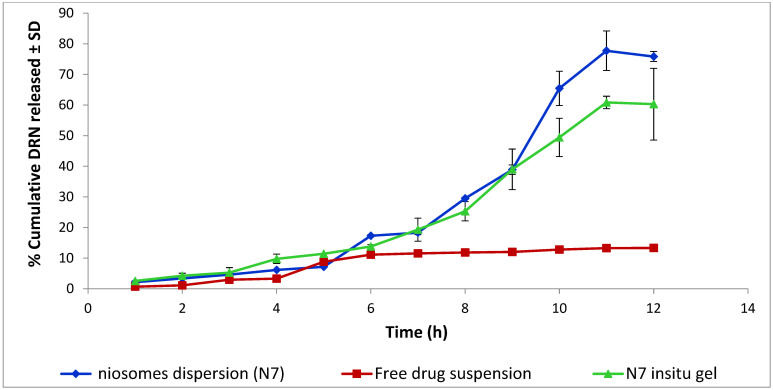
Release profile of DRN niosomes dispersion (N7), N7 in situ gel and free DRN suspension.

The drug release from the formulations will directly influence drug absorption at the nasal cavity. In vitro release profile showed enhanced cumulative% DRN released from both niosomal dispersion and niosomal in situ gel compared to drug-free suspension (Figure 5). Similarity factor (ƒ_2_) values of 25.02 for N7 and 31.07 for niosomal in situ gel compared to drug-free suspension were obtained. ƒ_2_ value below 50 indicated that the compared formulations were not equivalent. The release profile of DRN from niosomal dispersion exhibited a biphasic pattern with an initial slow drug release observed during the first 8 h, followed by a slightly rapid release pattern in the next 4 h. This pattern of release confirms the high entrapment efficiency of DRN niosomes. The vesicles were intact and stable enough to retard the rapid outflow of the drug. More than 75% DRN was released from niosomal dispersion (77.70 ± 1.02%) within 12 h compared to only 13.30 ± 1.20% released from drug-free suspension within the same period of time. The vast surface area of the formed nanovesicles, as well as the incorporation of surfactant (Sp 80) enhanced DRN diffusion from the prepared niosomes to the medium leading to a considerable increase in DRN released compared to the drug-free suspension. DRN niosomal in situ gel released 60.29 ± 11.30% within 12 h. This decrease in % of drugs released indicates that the gelling agent (gellan gum) has a delaying effect on the amount of DRN released. This effect may be due to acquired viscosity which acts as an additional diffusion barrier [36]. Unexpectedly, despite the apparent numerical difference in% release between niosomal dispersion and niosomal in situ gel, however, an *f*_2_ value of 53.64 implies that their dissolution profiles are comparable.

#### Release Mechanism of Optimal DRN Niosomal In Situ Gel

Upon comparing the regression coefficient values of different kinetic models, it was observed that the R^2^ value of different kinetic models and release data were found to best fit with Korsmeyer–Peppas model (Table 5). The Korsmeyer–Peppas model “power law” is a comprehensive model to depict drug release from polymeric systems [37]. This model describes the main transport phenomena involved in the release, either by diffusion or swelling [65]. The release exponent (n) was greater than 1; hence, the diffusional DRN release pattern from niosomal in situ gel agreed with non-Fickian (Super Case II transport), where drug release is predominately controlled by matrix relaxation and erosion [66]. This suggests that more than one mechanism may be involved in the release, i.e., drug release by diffusion, erosion, and polymer chain relaxation [37]. Super Case II transport is exhibited when diffusion and relaxation rates are equivalents [67]. The relaxational behavior of the polymer, accompanied by swelling and erosion, was attributed to the hydrophilic nature of the polysaccharide gelling agent (gellan), leading to super Case II transport [67]. Non-Fickian processes are normally found in vitreous (glassy) polymers when the environmental temperature is less than the vitreous transition temperature of the polymer (*T_g_*). A fast increase in the absorption rate of the solvent may sometimes occur. In this situation, the transport Super Case II takes place due to the expansion of forces exercised by swollen gel in the vitreous nucleus [68]. In Super Case II transport, the solvent diffusion is more rapid leading to rush of solvent penetration [69]. Finally, it is noteworthy that the simultaneous effect of more than one transport mechanism may occur in the same system, as reported by Klech and Simonelli [69].

### 3.6. In Vivo Pharmacokinetic Study

#### 3.6.1. Liquid Chromatography/Mass Spectrometry (LC-MS/MS) Bioanalytical Method for Determination of Dronedarone in Rabbits’ Plasma

No significant interferences with dronedarone or nitrofurantoin (IS) were observed in the chromatographed rabbits’ plasma used for the preparation of calibration standards and quality control samples. The recorded retention times of dronedarone and nitrofurantoin were 1.279 and 0.827 min, respectively. The overall chromatography time was 1.5 min. A standard plot obtained for plasma samples demonstrated high linearity in the range of 3–200 ng/mL with acceptable inter and intraday reproducibility.

#### 3.6.2. Experimental Observation and Estimated Pharmacokinetic Parameters

**Figure 6:**
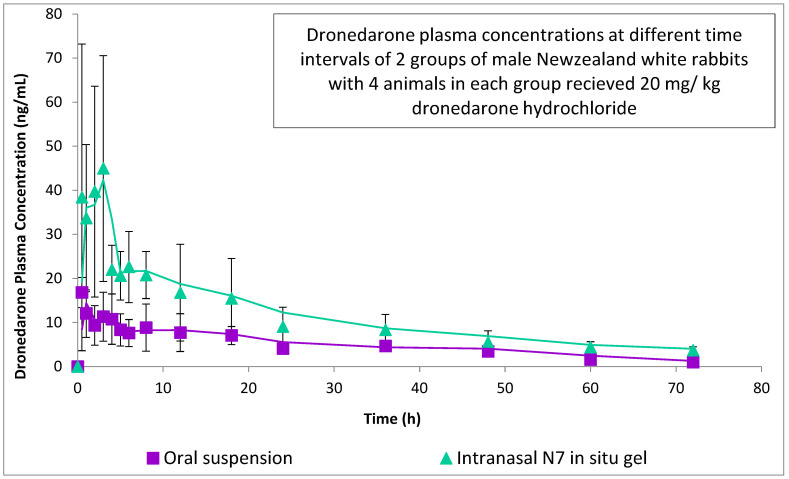
Plasma concentration-time curve of dronedarone after administration of intranasal N7 in situ gel and DRN oral suspension to experimental rabbits.

All the participating rabbits well tolerated the drug and the procedure implemented in the study. Every blood sample was obtained at the proper time. Plasma concentration—time data of dronedarone following oral administration of DRN suspension (treatment O) and intranasal administration of DRN niosomal in situ gel (treatment N) are shown in Figure 6. The pharmacokinetic parameters determined for the two treatments and the summary of these parameters are shown in Table 6.

#### 3.6.3. Statistical Analysis of Pharmacokinetic Parameters

ANOVA testing revealed that C_max_, AUC_0–72_, and AUC_0–∞_ of intranasal niosomal in situ gel were significantly higher than oral suspension (Table 6). The elevated value of C_max_ confirms the superior solubility, release extent, and absorption of DRN upon entrapment into niosomes. Time of peak plasma concentration (T_max_) of dronedarone after administration of treatment N was numerically higher than that of treatment O. However, statistical analysis of T_max_ using a non-parametric Mann–Whitney’s test showed a non-significant difference among both treatments (*p* = 0.1736). Prolonged T_max_ of the intranasal niosomal in situ gel, compared to an oral drug suspension, might be due to the extended release of DRN from the niosomes vesicles, as discussed previously [70]. Furthermore, lipid vesicles such as niosomes are characterized by protracted circulation time following their absorption; consequently, DRN is sheltered in the blood before its release, contrary to drug-free suspension [71]. The relative bioavailability of intranasal DRN niosomal in situ gel compared to the oral DRN suspension was found to be 195.72%. The high relative bioavailability value obtained substantiated the supremacy of niosomal intranasal in situ gel over oral suspension, which could be explained by several facts. Firstly, the instilled formulation was transformed into a gel when inserted into the nasal cavity, leading to prolongation of the residence time of the formulation on the nasal mucosa. This overcame the rapid mucociliary washout, which is considered the most common problem with the intranasal route. Secondly, the lipophilic nature of the niosomes allowed better vesicle penetration and more efficient drug absorption through the nasal mucosal membrane into the systemic circulation [15]. Moreover, efficient nanosizing of drug particles by entrapment into nanovesicular carriers and the presence of surfactants in vesicular arrangements also improved drug solubility and permeability and, finally, bypassed the liver first pass metabolism through intranasal delivery.

## 4. Conclusions

DRN was successfully entrapped into niosomal carriers, which demonstrated nano-size, narrow size distribution, high zeta potential, high drug encapsulation, and enhanced extent of DRN released compared to a drug-free suspension. The optimal niosomal formula (N7) was efficiently incorporated into the gellan polymeric matrix that exhibited sol to gel phase transition triggered by physiological ions present in nasal fluid. The formed gel showed good and acceptable rheology, gel strength, and mucoadhesiveness. Enhanced DRN release was attained from both N7 dispersions and in situ gel. In vivo pharmacokinetic assessment proved the superiority of niosomal in situ gelling sprays over oral suspension with respect to the rate and extent of DRN absorption, and an almost twofold increase in bioavailability was achieved. The found auspicious results of the current study verified the aptitude of the optimized niosomal in situ gel to be a promising intranasal delivery system for DRN that abolish its extensive first-pass metabolism upon oral administration.

## Figures and Tables

**Table 1:** The model summary statistics of 3^2^ full factorial design used for optimization of DRN loaded niosomes ^a^.

Independent Variables (Factors)	Constrains			
X1: Span^®^ type	Sp40	Sp60		Sp80
X2: Sp: CH ratio (*w*:*w*)	1:1	2:1		4:1
Dependent variables (Responses)	Constrains			
Y1:VS (nm)	Minimize			
Y2:PDI	Minimize			
Y3:ZP (mV)	Maximize			
Y4:EE%	Maximize			
Responses	Y1: VS (nm)	Y2: PDI	Y3: ZP (mV)	Y4: EE%
Minimum	100.90	0.23	4.24	40.52
Maximum	633.20	1.00	35.70	76.34
Ratio	6.28	4.27	8.42	1.8
Model	2FI	2FI	2FI	2FI
Adequate precision	62.44	11.97	13.75	17.86
R^2^	0.996	0.90	0.91	0.93
Adjusted R^2^	0.994	0.86	0.86	0.905
Predicted R^2^	0.991	0.78	0.79	0.85
Model *p*-value	<0.0001	<0.0001	<0.0001	<0.0001
Significant variables	X1, X2	X1, X2	X1	X1, X2

^a^ 2FI: two factor interactions.

**Table 2:** LC-MS/MS parameters selected for quantification of dronedarone using nitrofurantoin as internal standard.

Ion Source Settings											
Ion Source	Gas Temp. (°C)	Gas Flow (L/min)		Sheath Gas Temp (°C)		Sheath Gas Flow (L/min)	Nebulizer (psi)		Capillary (V)		Nozzle Voltage
ESI	300	10		300		10	50		4500		2000
**Scan Settings**											
Compound name		Scan Type	Polarity	Precursor ion	MS1 Res	Product Ion	MS2 Res	Dwell Time	Fragmentor	Collision Energy	Cell Accelerator Voltage
Dronedarone		MRM	+ve	557.2	Wide	100	Wide	200	120	25	3
Nitrofurantoin		MRM	−ve	237	Wide	151.9	Wide	300	100	18	3

**Table 3:** Independent variables and measured responses of 3^2^ full factorial design of DRN loaded niosomes.

Run No	Formula	Variables		Responses ± SD			
		X1: Span^®^ Type	X2: Sp:CH	Y1:VS (nm)	Y2: PDI	Y3: ZP (mV)	Y4: EE%
1	N1	Sp40	1:1	490.74 ± 16.43	1.00 ± 0.00	−9.62 ± 8.74	66.17 ± 1.63
2	N2	Sp40	2:1	227.77 ± 10.66	1.00 ± 0.00	−34.33 ± 0.03	57.66 ± 4.44
3	N3	Sp40	4:1	130.83 ± 5.50	0.61 ± 0.09	−21.00 ± 1.85	42.96 ± 2.58
4	N4	Sp60	1:1	607.64 ± 29.50	1.00 ± 0.00	−18.10 ± 1.91	64.63 ± 3.42
5	N5	Sp60	2:1	378.62 ± 8.30	0.59 ± 0.05	−16.70 ± 0.46	50.51 ± 3.20
6	N6	Sp60	4:1	154.64 ± 11.52	0.83 ± 0.30	−8.00 ± 1.54	64.55 ± 2.63
7	N7	Sp80	1:1	121.27 ± 13.31	0.43 ± 0.07	−22.23 ± 2.84	73.44 ± 2.80
8	N8	Sp80	2:1	113.65 ± 6.16	0.41 ± 0.04	−7.80 ± 1.20	68.80 ± 3.40
9	N9	Sp80	4:1	105.87 ± 6.71	0.25 ± 0.02	−26.70 ± 0.78	66.32 ± 1.30

**Table 4:** Effect of storage on physical stability of N7 ^a,b^.

Parameters	Freshly Prepared	After 90 Days at 4 °C	After 90 Days at 25 °C	*p*-Value
VS (nm)	121.27 ± 13.31	121.58 ± 12.89	125.38 ± 13.43	0.915
PDI	0.432 ± 0.07	0.42 ± 0.10	0.44 ± 0.05	0.919
ZP (mV)	−22.23 ± 2.80	−21.56± 2.09	−20.44 ± 1.89	0.652
EE%	73.44 ± 2.78	71.70± 2.79	70.99 ± 2.44	0.548

^a^ Data represented as mean ± SD (n = 3). ^b^ Difference is statistically significant at *p*-value < 0.05.

**Table 5:** Release kinetic models used to describe DRN release from optimal niosomal in situ gel.

Model	N7 In Situ Gel Regression Data	
	R²	n
Zero order	0.8985	
First order	0.8368	
Korsmeyer–Peppas	0.9515	1.45
Higuchi	0.7185	
Hixson Crowell	0.8591	

**Table 6:** Pharmacokinetic parameters of dronedarone following the administration of intranasal niosomal in situ gelling spray (treatment N) and oral suspension (treatment O) ^a,b^.

Parameters	Intranasal In Situ gel (Treatment N)	Oral Suspension (Treatment O)	*p*-Value
C_max_ (ng/mL)	57.99 ± 29.66	16.79 ± 3.42	0.033
T_max_ (h)	2.5	0.5	0.174
AUC_0–72_ (ng h/mL)	766.93 ± 251.74	320.76 ± 93.48	0.016
AUC_0–∞_ (ng h/mL)	919.73 ± 250.03	469.92 ± 107.25	0.016
t_1/2_ (h)	28.28 ± 11.03	31.25 ± 6.64	
K_el_ (h^−1^)	0.027 ± 0.008	0.023 ± 0.006	
% Relative bioavailability	195.72	-	

^a^ T_max_ expressed in median. All other values expressed in mean ± SD. ^b^ Oral suspension is used as reference standard. % Relative bioavailability = AUC_0–∞_intranasal in situ gel/AUC_0–∞_ oral suspension × 100.

## Data Availability

Not applicable.
